# Classical Density Functional Theory Coupled to the SAFT-VR Mie Equation of State: Extension to Associating Fluids

**DOI:** 10.1021/acs.jpcb.6c00630

**Published:** 2026-05-23

**Authors:** André de Freitas Gonçalves, Nathan Barros de Souza, Luis Fernando Mercier Franco

**Affiliations:** 28132 Universidade Estadual de Campinas (UNICAMP), Faculdade de Engenharia Química, Campinas CEP:13083-852, SP, Brazil

## Abstract

We have proposed a theoretical framework that enables the extension of the SAFT-VR Mie Equation of State to inhomogeneous associating fluids, on the basis of the weighted density formalism of classical density functional theory. A Helmholtz energy functional well established in the literature has been incorporated to represent the effect of associating interactions. For water confined in carbon slit-pores of few nanometers, the model predicts density profiles in good agreement with molecular simulations performed in the grand-canonical ensemble. Adsorption and desorption isotherms are obtained for different pore sizes, and capillary condensation and hysteresis are investigated by mapping the grand-potential. The results of our calculations indicate that the most stable curve generally matches one of the curves of GCMC, either adsorption or desorption. Further comparison with another theoretical approach available in the literature indicates similar performance of the models.

## Graphical abstract

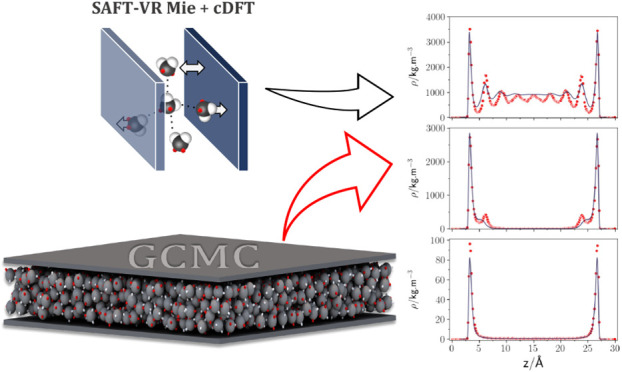

## Introduction

Statistical mechanics offer a convenient way to connect the structural properties of a system, on the molecular scale, to its macroscopic properties. Methods such as molecular simulations, integral-equation theories, and classical density functional theory (cDFT) provide different routes to establish such a connection. cDFT, in particular, offers a compromise between relative ease of implementation and detailed representation of molecular systems, despite its coarse-grained approach. It is a specially useful tool in the study of inhomogeneous systems, e.g., fluids near interfaces, or fluids in confining environments, such as the pores of an adsorbent. Yet, its applications are not limited to interfacial properties^1–3^ or adsorption,^4,5^ but also extend to research areas such as melting and freezing transitions,^6–8^ wetting,^9^ liquid crystals,^10,11^ and polymers,^12,13^ to mention some. With the recent advances in computational power and artificial intelligence, more realistic 3D-cDFT models have become more efficient,^14,15^ from the perspective of computational performance, and their use on screening and tailoring of complex porous materials, for example, are promising applications.^14,16,17^

In a concise manner, the main concept behind cDFT is that, for a fluid under the effect of an external potential, its intrinsic Helmholtz energy, i.e., the one arising from fluid–fluid molecular interactions, may be formulated as a functional of the density profile. Although alternative formulations in the canonical ensemble have already been proposed,^18,19^ the original and more usual theoretical development is set at constant volume (*V*), temperature (*T*) and chemical potential of all constituents (μ_*i*_), i.e., grand-canonical ensemble. As a consequence of the second law of thermodynamics, the grand-canonical functional reaches its global minimum at equilibrium. Thus, a variational principle arises, and the equilibrium density profile can be determined by minimizing the grand-canonical functional. Excellent reviews on the fundamentals of cDFT are available in the literature.^20–22^ More recently, a pedagogical review on basic concepts, numerical procedures, and available computational codes has also been proposed.^23^

One of the challenges of cDFT, and also the goal of many related studies, is the development of accurate and self-consistent Helmholtz energy functionals, more specifically, residual Helmholtz energy functionals. This can be done, for example, following a perturbative treatment, in which the residual contributions are further split into those from a reference system, and from the perturbed one. The Statistical Associating Fluid Theory (SAFT), originally developed for homogeneous systems, is a prominent example.^24,25^ Uncountable bulk Equations of State (EoSs) have been proposed on the basis of the SAFT formalism, with a variety of reference systems, including hard-chains,^26,27^ Lennard-Jones,^28,29^ Yukawa,^30,31^ and Mie^32–34^ fluids. The extension of the SAFT to inhomogeneous systems has also been explored in multiple studies. For systems with smooth density variations, e.g., bulk vapor–liquid interfaces, a common practice is to propose functionals for the reference fluid on the basis of the Local Density Approximation (LDA), so that the Helmholtz energy at a given coordinate is equal to that of a homogeneous fluid, evaluated at the corresponding local density.^21^ The studies from Llovel et al.^35^ and Gloor et al.^36^ fall into this category. The perturbation terms are often treated at the mean field level.^23^ On the other hand, systems with sharp density variations, e.g., fluids near hard-walls, or confined fluids, require a nonlocal treatment, usually performed on the basis of the Weighted Density Approximation (WDA). The Helmholtz energy of the reference system becomes a functional of weighted densities, which are, in turn, functionals of the local density, calculated as convolutions of the density with specific weight functions. Particularly for hard-sphere reference systems, the Fundamental Measure Theory (FMT)^37^ and its subsequent variations^38,39^ became the golden standard of such an approach. Belonging to this category, we may include the iSAFT model of Chapman and co-workers,^40–42^ the SAFT-FMT-DFT from Schindler et al.,^43^ the NLDFT/SAFT-VR from Malheiro et al.,^44,45^ and the PCP-SAFT from Sauer et al.,^2^ to mention a few.

For inhomogeneous associating fluids, the most common approaches are generally divided into two categories: one can start directly from an inhomogeneous formulation of Wertheim’s perturbation theory, as performed by Segura et al.,^46^ Chapman and co-workers,^40–42,47^ and more recently by Barthes et al.,^48,49^ or by extending the associative term of the bulk formulation to the inhomogeneous systems on the basis of the WDA formalism, as performed by Segura et al.,^46^ Yu and Wu,^50^ and Vergara et al.^51^ Although each of these models presents singularities, there is no ultimate approach to handling inhomogeneous associating fluids, and the performance of one over the others seems to depend on factors such as the extension of associative interactions and the degree of confinement.^48,51^ In a previous study, we proposed the extension of the SAFT-VR Mie EoS to inhomogeneous alkanes confined in slit pores on the basis of the WDA formalism.^52^ Here, we propose the inclusion of a Helmholtz energy functional due to the presence of associating interactions among fluid molecules. To do so, we incorporate the functional presented by Yu and Wu for the contribution due to association.^50^ Density profiles of water confined in carbon-slit pores are predicted with the theoretical model, and the results are further compared to the results of Monte Carlo simulations performed in the grand-canonical ensemble (GCMC).

## Theoretical Framework

Classical density functional theory establishes that, for a system at constant temperature (*T*), volume (*V*), and chemical potential of species *i* (μ_*i*_), the equilibrium density profile of each component (ρ_*i*_(**r**)), when subject to an external potential *V*_*i*_^ext^(**r**), is the one that minimizes the grand-canonical potential (Ω).^53^ The grand-canonical potential and intrinsic Helmholtz energy of the system ( F ) are both functionals of the density, and are related by

1 $$\Omega \left[\{\rho_{i}\left(r\right)\}\right]=F\left[\{\rho_{i}\left(r\right)\}\right]+\sum_{i=1}^{N_{c}}\int \rho_{i}\left(r\right)\left(V_{i}^{\mathrm{ext}}\left(r\right)-\mu_{i}\right)dr$$

where *N*_c_ is the number of components of the system, and the curly brackets represent the vector of molecular densities. Taking the derivative of eq 1 with respect to the density profile of component *j*, and setting the result equal to zero, the so-called Euler–Lagrange Equations are derived

2 $$\frac{\delta \left(F\left[\{\rho_{i}\left(r\right)\}\right]\right)}{\delta \rho_{j}\left(r\right)}+V_{j}^{\mathrm{ext}}\left(r\right)-\mu_{j}=0,,j=1,2,...,N_{c}$$

The intrinsic Helmholtz energy may be further split into ideal ( $F^{\mathrm{id}}$ ) and residual terms ( $F^{\mathrm{res}}$ )­

3 $$F=F^{\mathrm{ig}}+F^{\mathrm{res}}$$

While an exact solution is known for the ideal contribution

4 $$F^{\mathrm{ig}}\left[\{\rho_{i}\left(r\right)\}\right]=k_{B}T\sum_{i=1}^{N_{c}}\int \rho_{i}\left(r\right)\left[\ln\left(\rho_{i}\left(r\right)\Lambda_{i}^{3}\right)-1\right]dr$$

where *k*_B_ is the Boltzmann constant, and Λ_*i*_ is the thermal de Broglie wavelength of component *i*, an expression for the residual contribution relies on approximations. In this context, thermodynamic perturbation theories offer a convenient approach, whereby a system with known structural and thermodynamic properties is chosen as a reference (e.g., hard-sphere system) from which the properties of the real fluid can be determined. Equations of state derived from the SAFT, as mentioned earlier, are primary examples of such an approach. Within the formalism of the SAFT-VR Mie EoS,^33^ the residual intrinsic Helmholtz energy of a fluid is decoupled into contributions from a reference hard-sphere fluid ( $F^{\mathrm{hs}}$ ), the dispersive interactions among the Mie monomers ( $F^{\mathrm{disp}}$ ), the formation of molecular chains of Mie segments ( $F^{\mathrm{chain}}$ ), and the interactions between association sites on the segments ( $F^{\mathrm{assoc}}$ )­

5 $$F^{\mathrm{res}}\left[\{\rho_{i}\left(r\right)\}\right]=F^{\mathrm{hs}}\left[\{\rho_{i}\left(r\right)\}\right]+F^{\mathrm{disp}}\left[\{\rho_{i}\left(r\right)\}\right]+F^{\mathrm{chain}}\left[\{\rho_{i}\left(r\right)\}\right]+F^{\mathrm{assoc}}\left[\{\rho_{i}\left(r\right)\}\right]$$

Once the residual contribution to the intrinsic Helmholtz energy is determined, the equilibrium density profiles can be obtained by solving eq 6 iteratively

6 $$\rho_{j}\left(r\right)=\rho_{j}^{b}\exp\left(\beta \mu_{j}^{\mathrm{res},b}-\beta \frac{\delta F^{\mathrm{res}}\left[\{\rho_{i}\left(r\right)\}\right]}{\delta \rho_{j}\left(r\right)}-\beta V_{j}^{\mathrm{ext}}\left(r\right)\right)$$

where eq 4 was used for the ideal-gas term, and the chemical potential was replaced by its bulk counterpart (μ_*j*_^b^)­

7 $$\mu_{j}^{b}=\mu_{j}^{\mathrm{res},b}+k_{B}T\ln\left(\rho_{j}^{b}\Lambda_{j}^{3}\right)$$

given their equality overall the system, in the grand-canonical ensemble.

Thus, the main task consists of developing appropriate expressions for each of the terms of eq 5. Following our previous work,^52^ such a development is performed on the basis of the bulk SAFT-VR Mie EoS, further extended to inhomogeneous fluids using weighted densities. Since detailed mathematical development has been provided previously, the description of each contribution will be given shortly, with the exception of the associative contribution, not addressed before. Except for the associative contribution, the notation used when presenting the functionals and related equations is directly developed for mixtures, to keep consistency with our previous work. The formulation proposed for inhomogeneous fluids reduces to the bulk SAFT-VR Mie EoS, in the limit of homogeneous fluids.

### Hard-Sphere

The modified fundamental measure theory (MFMT),^38^ or White-Bear (WB) version of the FMT,^39^ derived from the Rosenfeld’s FMT,^37^ is used in the calculations, given its compatibility with the Boublík-Mansoori-Carnahan–Starling-Leland (BMCSL) EoS^54,55^ in the bulk limit, consistent with the SAFT-VR Mie description.^33^ The functional for the hard-sphere contribution is written as

8 $$\beta F^{\mathrm{hs}}\left(\{\rho_{i}\left(r\right)\}\right)=\int \Phi \left(\{n_{\alpha }\left(r\right)\}\right)dr$$

where Φ­({*n*_α_(**r**)}) is the Helmholtz energy density, a function of different weighted-densities

9 $$\Phi \left(\{n_{\alpha }\}\right)=-n_{0}\ln\left(1-n_{3}\right)+\frac{n_{1}n_{2}-n_{V1}\cdot n_{V2}}{\left(1-n_{3}\right)}+\frac{n_{3}+{\left(1-n_{3}\right)}^{2}\ln\left(1-n_{3}\right)}{36\pi n_{3}^{2}{\left(1-n_{3}\right)}^{2}}\times \left(n_{2}^{3}-3n_{2}n_{V2}\cdot n_{V2}\right)$$

each of them calculated as convolutions of the local density with weight functions (ω_*i*_^α^)­

10 $$n_{\alpha }\left(r\right)=\sum_{i=1}^{N_{c}}m_{i}\int \rho_{i}\left(r^{\prime }\right)\omega_{i}^{\alpha }\left(r-r^{\prime }\right)dr^{\prime }=\sum_{i=1}^{N_{c}}m_{i}\rho_{i}\left(r\right)⊗\omega_{i}^{\alpha }\left(r\right)$$

The weight functions of FMT are defined as

11a $$\omega_{i}^{0}\left(r\right)=\frac{1}{4\pi R_{i}^{2}}\delta \left(R_{i}-\left|r\right|\right)$$

11b $$\omega_{i}^{1}\left(r\right)=\frac{1}{4\pi R_{i}}\delta \left(R_{i}-\left|r\right|\right)$$

11c $$\omega_{i}^{2}\left(r\right)=\delta \left(R_{i}-\left|r\right|\right)$$

11d $$\omega_{i}^{3}\left(r\right)=\Theta \left(R_{i}-\left|r\right|\right)$$

11e $$\omega_{i}^{V1}\left(r\right)=\frac{1}{4\pi R_{i}}\frac{r}{\left|r\right|}\delta \left(R_{i}-\left|r\right|\right)$$

11f $$\omega_{i}^{V2}\left(r\right)=\frac{r}{\left|r\right|}\delta \left(R_{i}-\left|r\right|\right)$$

with δ and Θ being the Dirac delta and Heaviside functions, respectively. The radius of the effective hard-sphere system (*R*_*i*_) is related to the Mie interaction potential (ϕ_*i*,Mie_(*r*)) as follows^33^

12 $$R_{i}=\frac{1}{2}\int_{0}^{\sigma_{i}}\left[1-\exp\left(-\beta \phi_{i,\mathrm{Mie}}\left(r\right)\right)\right]dr$$

with ϕ_*i*,Mie_(*r*)­given by

13 $$\phi_{i,\mathrm{Mie}}\left(r\right)=\varepsilon_{i}\frac{\lambda_{r,i}}{\lambda_{r,i}-\lambda_{a,i}}{\left(\frac{\lambda_{r,i}}{\lambda_{a,i}}\right)}^{\lambda_{a,i}/\lambda_{r,i}-\lambda_{a,i}}\left[{\left(\frac{\sigma_{i}}{r}\right)}^{\lambda_{r,i}}-{\left(\frac{\sigma_{i}}{r}\right)}^{\lambda_{a,i}}\right]$$

where *r* is the distance between the centers of mass of the monomers, λ_*a*,*i*_ and λ_*r*,*i*_ are the attractive and repulsive exponents of the pair potential, respectively, σ_*i*_ is the diameter of the monomer, and ε_*i*_ is the depth of the potential. After applying the chain rule to eq 8, the derivative of the intrinsic Helmholtz energy functional with respect to the density profile of component *j* reads as

14 $$\frac{\delta \beta F^{\mathrm{hs}}\left[\{\rho_{i}\left(r\right)\}\right]}{\delta \rho_{j}\left(r\right)}=\int \sum_{\alpha }\frac{\partial \Phi }{\partial n_{\alpha }\left(r^{\prime }\right)}\frac{\delta n_{\alpha }\left(r^{\prime }\right)}{\delta \rho_{j}\left(r\right)}dr^{\prime }$$

Taking the derivatives of eqs 9 and ([Disp-formula eq10]), and replacing in eq 19, one can write the derivative of the hard-sphere functional as a sum of convolutions of the weighted densities with the derivatives of the Helmholtz energy density

15 $$\frac{\delta \left(\beta F^{\mathrm{hs}}\left[\{\rho_{i}\left(r\right)\}\right]\right)}{\delta \rho_{j}\left(r\right)}=m_{j}(\frac{\partial \Phi }{\partial n_{0}}⊗\omega_{j}^{0}+\frac{\partial \Phi }{\partial n_{1}}⊗\omega_{j}^{1}+\frac{\partial \Phi }{\partial n_{2}}⊗\omega_{j}^{2}+\frac{\partial \Phi }{\partial n_{3}}⊗\omega_{j}^{3}-\frac{\partial \Phi }{\partial n_{V1}}⊗\omega_{j}^{V1}-\frac{\partial \Phi }{\partial n_{V2}}⊗\omega_{j}^{V2})$$

### Dispersive Interactions

The Helmholtz energy functional related to the dispersive interactions reads as^1,2,52^

16 $$\beta F^{\mathrm{disp}}\left[\{\rho_{i}\left(r\right)\}\right]=\int {\mathrm{\rho ̅}}^{\mathrm{disp}}\left(r\right){\mathrm{a̅}}^{\mathrm{disp}}\left(\{{\mathrm{\rho ̅}}_{i}^{\mathrm{disp}}\left(r\right)\}\right)dr$$

where *a̅* ^disp^ is the reduced Helmholtz energy of the inhomogeneous system, which is itself a functional of the density profiles, and a function of the three perturbation terms of the Helmholtz energy (*a̅*_1_, *a̅*_2_, *a̅*_3_)­

17 $${\mathrm{a̅}}^{\mathrm{disp}}\left(\left\{\overset{®}{\rho_{i}\;}\left(r\right)\right\}\right)=\left(\sum_{i=1}^{N_{c}}{\mathrm{x̅}}_{i}m_{i}\right)\left({\mathrm{a̅}}_{1}+{\mathrm{a̅}}_{2}+{\mathrm{a̅}}_{3}\right)$$

The absolute weighted density (ρ̅^disp^) is defined as the sum of the individual ones,

18 $${\mathrm{\rho ̅}}^{\mathrm{disp}}\left(r\right)=\sum_{i=1}^{N_{c}}{\mathrm{\rho ̅}}_{i}^{\mathrm{disp}}\left(r\right)$$

and the mole fraction of component *i* (*x̅*_*i*_) is defined as

19 $${\mathrm{x̅}}_{i}=\frac{{\mathrm{\rho ̅}}_{i}^{\mathrm{disp}}\left(r\right)}{{\mathrm{\rho ̅}}^{\mathrm{disp}}\left(r\right)}$$

Each perturbation term of eq 22 retains the same meaning as its bulk counterpart, so the same equations in the core of the SAFT-VR Mie EoS apply here,^33^ except that the local density is replaced by the weighted density

20 $${\mathrm{\rho ̅}}_{i}^{\mathrm{disp}}\left(r\right)=\int \rho_{i}\left(r^{\prime }\right)\omega_{i}^{\mathrm{disp}}\left(r-r^{\prime }\right)dr^{\prime }$$

where the weight function (ω_*i*_^disp^) reads as

21 $$\omega_{i}^{\mathrm{disp}}\left(r\right)=\frac{3}{4\pi \psi_{i}^{3}d_{i}^{3}}\Theta \left(\psi_{i}d_{i}-\left|r\right|\right)$$

with *d*_*i*_ = 2*R*_*i*_. The parameter ψ_*i*_ is computed from the perturbative part of the Mie interaction potential^52,56^

22 $$\psi_{i}={\left[\frac{\lambda_{r,i}}{\lambda_{r,i}-\lambda_{a,i}}{\left(\frac{\lambda_{r,i}}{\lambda_{a,i}}\right)}^{\lambda_{a,i}/\lambda_{r,i}-\lambda_{a,i}}\left(\frac{3}{\lambda_{a,i}-3}-\frac{3}{\lambda_{r,i}-3}\right)\right]}^{1/3}$$

The derivative of eq 21 with respect to the density profile of component *j* may be written as a convolution

23 $$\frac{\delta \left(\beta F^{\mathrm{disp}}\left[\left\{\rho_{i}\left(r\right)\right\}\right]\right)}{\delta \rho_{j}\left(r\right)}=\left(\frac{\partial \left[{\mathrm{\rho ̅}}^{\mathrm{disp}}\left(r\right){\mathrm{a̅}}^{\mathrm{disp}}\left(\{{\mathrm{\rho ̅}}_{i}^{\mathrm{disp}}\left(r\right)\}\right)\right]}{\partial {\mathrm{\rho ̅}}_{i}^{\mathrm{disp}}\left(r\right)}\right)⊗\omega_{j}^{\mathrm{disp}}\left(r\right)$$

### Molecular Chain

The free-energy functional resulting from the formation of chains of Mie monomers is also adapted on the basis of the WDA formalism^52^

24 $$\beta F^{\mathrm{chain}}\left[\{\rho_{i}\left(r\right)\}\right]=\int \rho \left(r\right){\mathrm{a̅}}^{\mathrm{chain}}\left(\{{\mathrm{\rho ̅}}_{i}^{\mathrm{chain}}\left(r\right)\}\right)dr$$

where the weighted density (ρ̅_*i*_^chain^) reads as

25 $${\mathrm{\rho ̅}}_{i}^{\mathrm{chain}}\left(r\right)=\int \rho_{i}\left(r^{\prime }\right)\omega_{i}^{\mathrm{chain}}\left(r-r^{\prime }\right)dr^{\prime }$$

with the chain weight function given by

26 $$\omega_{i}^{\mathrm{chain}}\left(r\right)=\frac{3}{4\pi d_{i}^{3}}\Theta \left(d_{i}-\left|r\right|\right)$$

From the definition of the reduced Helmholtz energy associated with chain formation, eq 29 is rewritten as

27 $$\beta F^{\mathrm{chain}}\left[\{\rho_{i}\left(r\right)\}\right]=-\sum_{i=1}^{N_{c}}\int \rho_{i}\left(r\right)\left(m_{i}-1\right)\ln{\mathrm{g̅}}_{\mathrm{ii}}^{\mathrm{Mie}}\left(\sigma_{\mathrm{ii}}\right)dr$$

where *g̅*_*ii*_^Mie^ is the radial distribution function of the inhomogeneous Mie fluid evaluated at contact. The derivative of eq 32 with respect to the density profile of component *j* reads as

28 $$\frac{\delta \left(\beta F^{\mathrm{chain}}\left[\{\rho_{i}\left(r\right)\}\right]\right)}{\delta \rho_{j}\left(r\right)}=-\left(m_{j}-1\right)\ln\left({\mathrm{g̅}}_{\mathrm{jj}}^{\mathrm{Mie}}\left(\sigma_{\mathrm{jj}}\right)\right)-\sum_{i=1}^{N_{c}}\left(\left(m_{i}-1\right)\frac{\rho_{i}\left(r\right)}{{\mathrm{g̅}}_{\mathrm{ii}}^{\mathrm{Mie}}\left(\sigma_{\mathrm{ii}}\right)}\frac{\partial {\mathrm{g̅}}_{\mathrm{ii}}^{\mathrm{Mie}}\left(\sigma_{\mathrm{ii}}\right)}{\partial {\mathrm{\rho ̅}}_{j}^{\mathrm{chain}}\left(r\right)}\right)⊗\omega_{j}^{\mathrm{chain}}\left(r\right)$$

### Associating Interactions

The development of a contribution term to the Helmholtz energy due to associating interactions follows Wertheim’s TPT1 treatment applied to the reference Mie fluid, as performed by Lafitte et al.^33^ For *N* molecules of a single component bulk fluid, such contribution is

29 $$\beta F^{\mathrm{assoc}}=N\sum_{a=1}^{s}n_{a}\left[\ln\chi_{a}-\frac{1}{2}\chi_{a}+\frac{1}{2}\right]$$

or equivalently, the Helmholtz energy density is

30 $$\beta \Phi^{\mathrm{assoc}}=\rho_{b}\sum_{a=1}^{s}n_{a}\left[\ln\chi_{a}-\frac{1}{2}\chi_{a}+\frac{1}{2}\right]$$

where ρ_*b*_ is bulk number density, and *n*_*a*_ and χ_*a*_ are, respectively, the number of sites of type *a* per segment and the fraction of nonbonded sites of type *a*, computed as

31 $$\chi_{a}=\frac{1}{1+\rho_{b}\sum_{b=1}^{s}n_{b}\chi_{b}\Delta_{\mathrm{ab}}}$$

The strength of associating interactions (Δ_*ab*_) is determined from the RDF of the reference Mie fluid, *g* ^Mie^(*r*), and from the angle average of the Mayer function related to the square-well potential that characterizes the associating interactions (ϕ_*ab*_(**r**_*ab*_)),^57^ as follows

32 $$\Delta_{\mathrm{ab}}=\int g^{\mathrm{Mie}}\left(r\right)\left\langle \exp\left(-\beta \phi_{\mathrm{ab}}\left(r_{\mathrm{ab}}\right)\right)-1\right\rangle dr$$

where

33 $$\varphi_{\mathrm{ab}}\left(r_{\mathrm{ab}}\right)=\{\begin{array}{ll} -\varepsilon_{\mathrm{ab}}^{\mathrm{HB}}, & \left|r_{\mathrm{ab}}\right|\leq r_{\mathrm{ab}}^{c},\\ 0, & \left|r_{\mathrm{ab}}\right|>r_{\mathrm{ab}}^{c} \end{array}$$

with **r**_*ab*_, *r*_*ab*_^*c*^, and ε_*ab*_^HB^ being, respectively, the distance between the association sites of types *a* and *b*, the cutoff radius, and the depth of the square-well potential. Equation 37 is more commonly presented in a factorized form as

34 $$\Delta_{\mathrm{ab}}=\sigma^{3}\left[\exp\left(\beta \varepsilon_{\mathrm{ab}}^{\mathrm{HB}}\right)-1\right]I_{\mathrm{ab}}$$

where *I*_*ab*_ is the association kernel

35 $$I_{\mathrm{ab}}=\frac{\pi }{6\sigma^{3}{r_{\mathrm{ab}}^{d}}^{2}}\int_{2r_{\mathrm{ab}}^{d}-r_{\mathrm{ab}}^{c}}^{2r_{\mathrm{ab}}^{d}+r_{\mathrm{ab}}^{c}}g^{\mathrm{Mie}}\left(r\right){\left(r_{\mathrm{ab}}^{c}+2r_{\mathrm{ab}}^{d}-r\right)}^{2}\left(2r_{\mathrm{ab}}^{c}-2r_{\mathrm{ab}}^{d}+r\right)rdr$$

where *r*_*ab*_^*d*^ is the distance between the center of the Mie segments and the association sites. Solving eq 40 requires the evaluation of the RDF of the Mie fluid over a range of distances between the segments, which is generally accomplished with the use of integral-equation theories, or with the aid of molecular simulations. Dufal et al.^32^ compared different approaches for deriving an algebraic expression for Δ_*ab*_, including an approximation of *g* ^Mie^(*r*) in terms of the contact value of the RDF of hard-spheres of diameter *d* - approach previously followed by Lafitte et al.^33^, the correlation of structural data of a Lennard-Jones fluid, obtained through molecular simulations,^28^ and the use of the reference hyper-netted chain (RHNC) integral equation theory to calculate the RDF of the Mie fluid, whereby the association kernel is numerically evaluated and further correlated with an empirical polynomial for a range of temperatures and densities.^32^ The last approach is reported to be the most accurate among the three. Nevertheless, it is not trivial to incorporate the formalism of WDA within such a method, essentially because of the use of correlations to the temperature and density proposed for the bulk fluid, which is also the case of the second approach, i.e., the LJ fluid. Also, the factorization of the association kernel through the RDF of a hard-sphere fluid has already been extended to inhomogeneous fluids through the incorporation of FMT densities,^50^ being tested in multiple studies, generally performing well.^2,48,51^ Here, we follow such an approach, as will be detailed soon. For a bulk fluid, the association kernel reads as

36 $$I_{\mathrm{ab}}=g_{d}^{\mathrm{HS}}\left(d\right)\kappa_{\mathrm{ab}}$$

with the bonding volume being

37 $$\kappa_{\mathrm{ab}}=4\pi d^{2}\left[\ln\left(\left(r_{\mathrm{ab}}^{c}+2r_{\mathrm{ab}}^{d}\right)/d\right)\left(6{r_{\mathrm{ab}}^{c}}^{3}+18{r_{\mathrm{ab}}^{c}}^{2}r_{\mathrm{ab}}^{d}-24{r_{\mathrm{ab}}^{d}}^{3}\right)+\left(r_{\mathrm{ab}}^{c}+2r_{\mathrm{ab}}^{d}-d\right)\left(22{r_{\mathrm{ab}}^{d}}^{2}-5r_{\mathrm{ab}}^{c}r_{\mathrm{ab}}^{d}-7r_{\mathrm{ab}}^{d}d-8{r_{\mathrm{ab}}^{c}}^{2}+r_{\mathrm{ab}}^{c}d+d^{2}\right)\right]/\left(72{r_{\mathrm{ab}}^{d}}^{2}\sigma^{3}\right)$$

and the RDF of the hard-sphere fluid evaluated at contact is given by^54^

38 $$g_{d}^{\mathrm{HS}}\left(d\right)=\frac{1}{1-\xi_{3}}+\frac{3}{2}d\frac{\xi_{2}}{{\left(1-\xi_{3}\right)}^{2}}+\frac{d^{2}}{2}\frac{\xi_{2}^{2}}{{\left(1-\xi_{3}\right)}^{3}}$$

The moments of the number density, ξ_2_ and ξ_3_, are given by

39 $$\xi_{l}=\frac{\pi }{6}d^{l}\rho_{s},,l=2,3$$

where the segment number density (ρ_*s*_) is the product of the molecular number density and the number of segments per molecule, i.e., ρ_*s*_ = *m*ρ. For an inhomogeneous fluid, Yu and Wu^50^ propose the replacement of ξ_2_, ξ_2_^2^, ξ_3_, and ρ_*b*_ in eqs 35, ([Disp-formula eq36]), and ([Disp-formula eq43]) with the weighted densities of FMT: $\frac{1}{6}n_{2}\zeta$ , $\frac{1}{36}n_{2}^{2}\zeta$ , *n*_3_ and *n*_0_ ζ, respectively, where ζ is a factor that incorporates the vector-weighted densities

40 $$\zeta =1-\frac{n_{V2}\cdot n_{V2}}{n_{2}^{2}}$$

The RDF for the inhomogeneous hard-sphere fluid becomes

41 $$g_{d}^{\mathrm{HS}}\left(d,n_{\alpha }\right)=\frac{1}{1-n_{3}}+\left(\frac{d}{4}\right)\frac{n_{2}\zeta }{{\left(1-n_{3}\right)}^{2}}+{\left(\frac{d}{2}\right)}^{2}\frac{n_{2}^{2}\zeta }{18{\left(1-n_{3}\right)}^{3}}$$

and the Helmholtz energy functional due to the associative interactions reads as

42 $$\beta F^{\mathrm{assoc}}\left[\rho \left(r\right)\right]=\int n_{0}\left(r\right)\zeta \left(r\right)\sum_{a=1}^{s}n_{a}\left[\ln\chi_{a}\left(r\right)-\frac{1}{2}\chi_{a}\left(r\right)+\frac{1}{2}\right]dr$$

with χ_*a*_(**r**) computed as

43 $$\chi_{a}\left(r\right)=\frac{1}{1+n_{0}\left(r\right)\zeta \left(r\right)\sum_{b=1}^{s}n_{b}\chi_{b}\left(r\right)\Delta_{\mathrm{ab}}\left(r\right)}$$

Since water has the same number of association sites of each type, with a single value of association strength, it follows from eq 48 that χ_*a*_(**r**) = χ_*b*_(**r**) = χ­(**r**). Thus, eq 48 can be solved explicitly for the fraction of nonbonded sites, χ­(**r**) as^45,58^

44 $$\chi \left(r\right)=\frac{-1+\sqrt{1+8n_{0}\left(r\right)\zeta \left(r\right)\Delta_{\mathrm{ab}}\left(r\right)}}{4n_{0}\left(r\right)\zeta \left(r\right)\Delta_{\mathrm{ab}}\left(r\right)}$$

Equations 39 and ([Disp-formula eq41]) are used in the calculation of Δ_*ab*_(**r**), with the RDF of the hard-sphere fluid being computed with eq 46.

## Methods

The capability of the SAFT-VR Mie-cDFT model is evaluated in two stages: initially, the density profiles of water adsorbed on carbon-slit pores of different wall-to-wall distances—7, 8, 14, and 30 Å—are compared to the results of GCMC simulations. The external field is represented by a 10–4–3 interaction potential (or Steele potential)^59^

45 $$\phi_{\mathrm{Steele}}\left(z\right)=2\pi \rho_{\mathrm{sol}}\varepsilon_{\mathrm{cross}}\sigma_{\mathrm{cross}}^{2}\Delta \left[\frac{2}{5}{\left(\frac{\sigma_{\mathrm{cross}}}{z}\right)}^{10}-{\left(\frac{\sigma_{\mathrm{cross}}}{z}\right)}^{4}-\frac{\sigma_{\mathrm{cross}}^{4}}{3\Delta {\left(z+\alpha \Delta \right)}^{3}}\right]$$

where ρ_sol_ is the solid density, Δ is the interlayer spacing, and *z* is the axial distance between the center of mass of the segment and the pore wall. The adjustable parameter α was set to 0.61 to improve the representation of the potential.^59^

In eq 50, σ_cross_ and *ε*_cross_ are the solid–fluid cross-interaction parameters, determined using the Lorentz–Berthelot combining rules^60,61^

46a $$\sigma_{\mathrm{cross}}=\frac{\sigma_{i}+\sigma_{\mathrm{sol}}}{2}$$

46b $$\varepsilon_{\mathrm{cross}}=\sqrt{\varepsilon_{i}\varepsilon_{\mathrm{sol}}}$$

where σ_sol_ is the size parameter of the solid (graphite), and ε_sol_ is the energy parameter.

Thermodynamic consistency between both implementations is guaranteed by, initially, applying the methodology previously described to a bulk fluid of known density, so that the bulk chemical potential can be determined as the derivative of the total intrinsic Helmholtz energy functional. This procedure is equivalent to setting *V*_*j*_^ext^(**r**) equal to zero in eq 6, and subsequently adding the ideal-gas term to the residual chemical potential. The computed chemical potential is then set as input for the GCMC simulations. In addition, the results of the theoretical model are compared to other set of results for similar models available in the literature. In all cases, an adapted Picard scheme^52^ is used for solving eq 6 iteratively for the density profile. Given the slit-like geometry, with the external field imposed by solid-walls acting along the *z*-direction, the implementation is carried out for a single dimension (*z*-coordinate). In the theoretical calculations, a grid with 8192 evenly spaced slabs is used for representing the slit pore, based on the discretization scheme proposed by Stierle et al.^14^ The convolutions are solved in the Fourier space, using the fast Fourier transform (FFT) code of Press et al.^62^ Further details on the discretization, analytical Fourier transforms of weight functions, and numerical procedures are provided in the Supporting Information of de Freitas Gonçalves et al.^52^

The codes for cDFT calculations and GCMC simulations are publicly available at the GitHub repository https://github.com/LESC-Unicamp/Supplementary-materials, under the MIT License.

### GCMC Simulation and Force Fields

Grand canonical Monte Carlo (GCMC) simulations are performed with our in-house code to validate the cDFT calculations for associating fluids confined in slit pores of different widths. All simulations are carried out at a fixed temperature of *T* = 425 K and equilibrated under a reduced chemical potential μ* = μ/(*k*_B_ *T*). The thermal de Broglie wavelength used in the GCMC simulations is Λ ≈ 0.19952 Å.

Density profiles are obtained by discretizing the simulation box along the *z*-direction (perpendicular to the confining walls) using a uniform grid consisting of 200 slabs. A value of μ* = −16.506 is used for slit pores with widths of 7 Å and 8 Å, μ* = −14.437 for the 14 Å pore, and μ* = −14.364 for the 30 Å pore. Initially, *N* = 500 water molecules are randomly placed in an orthorhombic simulation box with a square cross section parallel to the confining walls. The initial system density (ρ = *N*/*V*) is chosen for each pore width to promote equilibration toward a comparable average number of molecules. Specifically, ρ is set to 0.003 Å^–3^, 0.002 Å^–3^, 0.0044 Å^–3^, and 0.00175 Å^–3^ for the pore widths of 7 Å, 8 Å, 14 Å, and 30 Å, respectively. Under these initial conditions, all systems reach equilibrium with an average number of approximately 2000 molecules.

Adsorption/desorption isotherms (ρ versus μ*) are obtained from simulations performed over the following chemical potential ranges: μ* ∈ [−20,–16.25] for slit pores with a width of 7 Å, μ* ∈ [−21,–15] for pores with 8 Å, μ* ∈ [−19,–14] for pores with 14 Å, and μ* ∈ [−18,–13.5] for pores with 30 Å. The upper bounds correspond to the starting points of desorption simulations (high-density states), whereas the lower bounds correspond to those of adsorption simulations (low-density states). The box dimensions and initial system densities are selected to ensure that the equilibrium configurations contain approximately between 100 and 4000 particles (see Table 1 for details). For each pore width *H*, ρ_ads_^ini^ and ρ_des_^ini^ are the initial densities for the adsorption and desorption simulations, respectively, while *L*_ads_ and *L*_des_ are the side lengths of the square cross sections of the corresponding simulation boxes.

**Table 1:** GCMC Simulation Parameters Used to Compute the Adsorption and Desorption Isotherms for Each Pore Width *H* ^a^

*H* (Å)	ρ_ads_^ini^ (Å^–3^)	*L*_ads_ (Å)	ρ_des_^ini^ (Å^–3^)	*L*_des_ (Å)
7	0.00137	144.150	0.011008	144.15
8	0.0000175	845.154	0.011341	213.201
14	0.000015	690.066	0.01725	97.590
30	0.000015	333.333	0.020114	72.822

a The initial densities correspond to the starting states of the adsorption (ρ_ads_^ini^) and desorption (ρ_des_^ini^) simulations. *L*_ads_ and *L*_des_ are the side lengths of the square cross-section of the simulation box for adsorption and desorption, respectively.

GCMC simulations are executed over 100 million Monte Carlo cycles, equally divided between equilibration and production stages. The Monte Carlo moves include: (i) translation of the center of mass of a water molecule, (ii) rotation of a water molecule about its center of mass, resulting in the displacement of its association sites, (iii) insertion of a water molecule into the system, and (iv) deletion of a water molecule from the system. All move types are attempted with equal probability. Further details regarding the implementation of these moves are provided in the Supporting Information.

The maximum translational (Δ*r*_max_) and rotational (Δθ_max_) displacements are initially set to 0.4σ_*i*_ and 0.1 rad, respectively. During the equilibration stage, these parameters are dynamically adjusted every 500 Monte Carlo cycles by ±5% of their current values to achieve an acceptance ratio of 50% for both translational and rotational moves. Rotational moves are implemented using quaternion algebra.^63^

After each Monte Carlo move, the fluid–fluid and solid–fluid interactions are evaluated such that the potential energy of a molecule *p* is given by

47 $$\phi_{p}=\phi_{\mathrm{wall}}\left(z_{p}\right)+\sum_{k\neq p}^{N}\left[\phi_{\mathrm{Mie}}\left(r_{\mathrm{pk}}\right)+\sum_{a=1}^{n_{\mathrm{sites}}}\sum_{b=1}^{n_{\mathrm{sites}}}\phi_{\mathrm{ab}}^{\left(p,k\right)}\left(r_{\mathrm{ab}}^{\left(p,k\right)}\right)\right]$$

where *r*_*pk*_ is the distance between the centers of mass of molecules *p* and *k*, *n*_sites_ is the number of association sites per water molecule, **r**_*ab*_ is the vector distance between association site *a* of molecule *p* and association site *b* of molecule *k*, and *z*_*p*_ is the axial distance between molecule *p* and the confining walls.

Each term in eq 53 represents a specific interaction contribution under the assumption of a spherical geometry for the water molecule. The term ϕ_Mie_ accounts for the dispersive intermolecular interactions (London dispersion forces), ϕ_wall_ accounts for the solid–fluid interactions, and ϕ_*ab*_^(*p*,*k*)^ accounts for the associating intermolecular interactions (hydrogen bonding).

Dispersive fluid–fluid interactions are described using a Mie potential—eq 18—with parameters taken from ref 32: ε_*i*_/*k*_B_ = 488.75 K, σ_*i*_ = 3.161 Å, λ_*a*,*i*_ = 6, and λ_*r*,*i*_ = 52.367.

Given the finite size of the simulated systems and the continuous nature of the Mie potential, a cutoff radius of *r*_Mie_^*c*^ = 28 Å is employed in the GCMC simulations. Pairwise interactions between molecules separated by distances *r*_*pk*_ > *r*_Mie_^*c*^ are neglected. Long-range (tail) corrections are not applied, since the contribution of interactions beyond this cutoff is negligible under the conditions considered.

Solid–fluid interactions are described using the Steele (10–4–3) potential—eq 50—with parameters taken from ref 59: ρ_sol_ = 0.114 Å^–3^, Δ = 3.35 Å, σ_sol_ = 3.4 Å, and ε_sol_/*k*_B_ = 28 K.

In the GCMC simulations, the simulation box is centered at the origin of the coordinate system such that *z*_*p*_ ∈ [−*H*/2, *H*/2], where *H* is the pore width. Accordingly, the coordinate must be first transformed as *z*_*p*_ → *z*_*p*_+*H*/2 prior to evaluating the Steele potential. Furthermore, the solid–fluid interaction is formulated for two parallel planar walls located at *z* = 0 and *z* = *H*, confining the fluid to the region 0 < *z* < *H*. The total external potential acting on a fluid molecule is therefore obtained as the sum of the contributions from each wall

48 $$V^{\mathrm{ext}}\left(z_{p}\right)\equiv \phi_{\mathrm{wall}}\left(z_{p}\right)=\phi_{\mathrm{Steele}}\left(z_{p}\right)+\phi_{\mathrm{Steele}}\left(H-z_{p}\right)$$

During the GCMC simulations, any trial move that results in an overlap between a molecule and the pore walls is immediately rejected. For a spherical molecular geometry, the overlap with a planar wall occurs when: (1) $z_{p}\leq \frac{\sigma_{i}}{2}$ or (2) $z_{p}\geq H-\frac{\sigma_{i}}{2}$ .

Associating interactions are characterized by representing the water molecule with a four-site association model,^64^ consisting of two hydrogen (H) sites and two lone-pair electron (E) sites. Association is allowed only between an H site on one molecule and an E site on another molecule. Interactions between identical sites (H–H or E–E) are not considered, and are therefore excluded from the simulations.

In the four-site model, each association site is positioned within a sphere of diameter σ_*i*_ at a distance *r*_*d*_ = 0.4σ_*i*_ from the molecular center of mass,^32,33^ forming a distorted tetrahedral arrangement such that the angles between the two H sites and between the two E sites are θ_site_ = 104.5° (see Figure 1).

**Figure 1:**
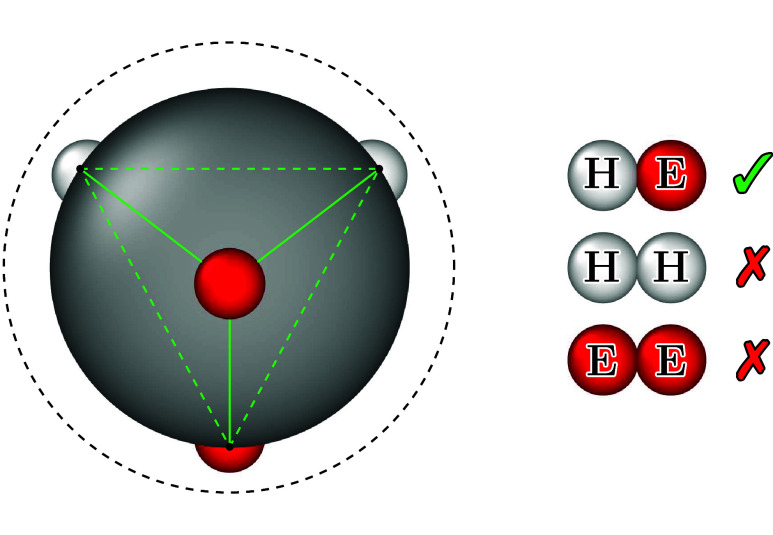
Illustration of the four-site association model for the water molecule. Hydrogen (H) sites are shown in white, while the oxygen lone-pair (E) sites are shown in red. The association sites are positioned on the surface of a sphere of radius *r*_*d*_, forming a distorted tetrahedral arrangement (highlighted in green). The black dashed outline represents the spherical geometry of the water molecule with diameter σ_*i*_. The right panel depicts the site–site interactions (hydrogen bonding) that are permitted and forbidden within the four-site model.

Associating interactions are described using a square-well (SW) potential—eq 38—with parameter ε_*ab*_^HB^/*k*_B_ = 1210 K taken from ref 32. The SW potential is evaluated only for site–site separation distances smaller than a cutoff radius of *r*_*ab*_^*c*^ = 0.5834σ_*i*_.

Interactions are evaluated between all pairs of association sites on the two molecules, resulting in a total of 16 site–site interaction checks per molecular pair. The four-site model allows at most two associations between a given pair of molecules, with each association site restricted to a single bond. Thus, a bonded site cannot associate with another molecule unless the existing bond is first disrupted during a Monte Carlo trial move.

During translational and rotational trial moves of a molecule *p* that is initially associated with a molecule *k*, the separation distance between the bonded sites is continuously monitored. When this distance exceeds the square-well cutoff, the association is disrupted. Upon bond dissociation, two checks are done: (1) whether the freed association site on molecule *p* can form a new valid association with neighboring sites on other molecules, and (2) whether the freed association site on molecule *k* can form a new valid association with neighboring sites. The latter is also performed during deletion trial moves, as the removal of a molecule *p* necessarily disrupts any existing association with *k*. Insertion trial moves do not include these reassociation checks.

## Results and Discussion

A comparison of cDFT and GCMC results for slit-pores of 7, 8, 14, and 30 Å width is presented in Figure 2. The bulk densities are set equal to 0.2566 kg·m^–3^ (Figure 2A–2C), and 2.267 kg·m^–3^ (Figure 2D), and the temperature is equal to 425 K, in all cases. In Figure 2B,C, the deviations between cDFT and GCMC results are concentrated mainly close to the walls.

**Figure 2:**
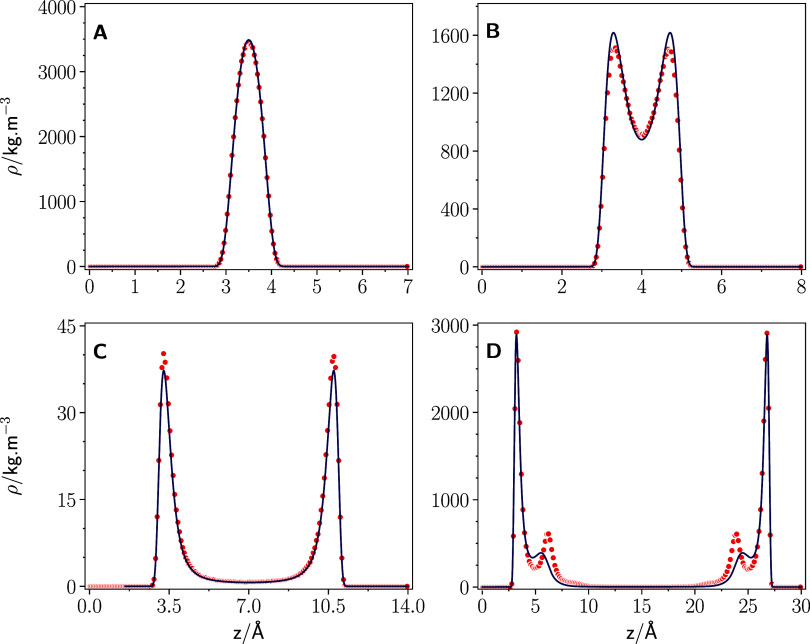
Density profiles of water on carbon slit-pores of (A) 7 Å, (B) 8 Å, (C) 14 Å, and (D) 30 Å width, at 425 K. The bulk density is equal to 2.267 kg·m^–3^ on (D), and 0.2566 kg·m^–3^ in the other cases. Full lines: SAFT-VR Mie - cDFT model; Circles: GCMC results.

For the cases illustrated in Figure 2A,D, the agreement observed for the highest peaks is more pronounced. Such cases correspond to the highest density peaks among the four scenarios illustrated here. Particularly for Figure 2D, we attribute most of the deviations between the models to the approximations inherent to cDFT, more specifically, to the modeling of the contributions related to the hard-sphere fluid and to the associating interactions. Vergara et al.,^51^ who have used the same MFMT-based functionals for hard-spheres and associating contributions as the ones used in this work, observed that the cDFT profiles of associative hard-spheres near hard-walls are often smoother than simulations results, as indicated in Figure 2D. The broad range of densities of the adsorbed fluid suggests that capillary condensation might occur at some point during the pore filling. To shed some light on the phase behavior of adsorbed water, adsorption isotherms at 425 K were obtained considering the pore sizes represented in Figure 2. The adsorption/desorption isotherms are shown in Figure 3, while the corresponding grand-potential is shown in Figure 4. For all pore sizes, cDFT predicts the occurrence of capillary condensation, along with hysteresis phenomenon. GCMC, however, reveal the occurrence of hysteresis only on the 14 Å and 30 Å pores (Figure 3C,D, respectively). The dashed vertical line in Figure 3 indicates the expected locus of the phase transitions, i.e., the corresponding chemical potential for which vapor and liquid coexist, and the grand-potential is at its minimum, as indicated in Figure 4. For Figure 3A,B, the most stable curves correspond to the adsorption process, while on Figure 3C,3D, they oscillate between the adsorption–desorption curves. The prediction of capillary condensation on the 7 Å and 8 Å pores might be related to the assumptions inherent to each model. Although water is represented with the same molecular structure in both cDFT and GCMC—a spherical segment with four association sites, the approximations underlying the weighted density formalism will naturally lead to different results, when compared to GCMC. This might explain why cDFT also predicts a first-order phase transition in the adsorption isotherm of Figure 3D, at a chemical potential of approximately −14.5, while GCMC simulations indicate a smoother transition. Multiple steps in adsorption–desorption curves exhibiting hysteresis have been already reported in the literature.^65^ Despite the differences in the representation of the phenomenon, both cDFT and GCMC indicates that it might be related to the formation of a second adsorption layer, as shown in Figure 5 (second density profile from the top). As the chemical potential increases further, a second phase transition is predicted by both models, although the chemical potentials corresponding to this transition is different (−14.25, for cDFT, and −14, for GCMC). Once the transition is completed, the entire pore is filled with a liquid-like phase, as shown in the first profile (top-right) of Figure 5. For cDFT, this transition occurs at the chemical potential of the bulk vapor–liquid transition (Supporting Information).

**Figure 3:**
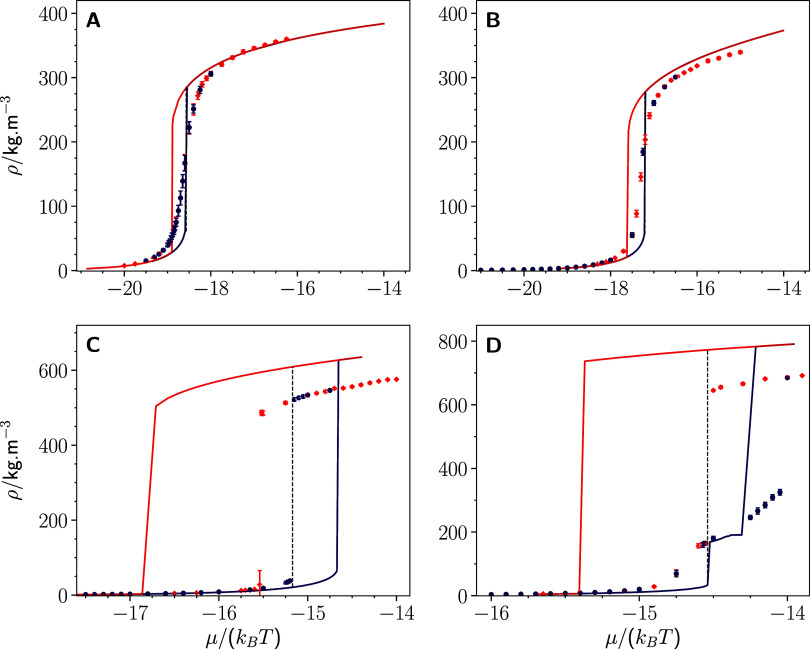
Adsorption isotherms of water on carbon slit-pores of (A) 7 Å, (B) 8 Å, (C) 14 Å, and (D) 30 Å width, at 425 K. The blue color represents adsorption curves, while red represents desorption curves. The black dashed line indicates the locus of the phase transition. Full lines: SAFT-VR Mie - cDFT model; Symbols with error bars: GCMC results.

**Figure 4:**
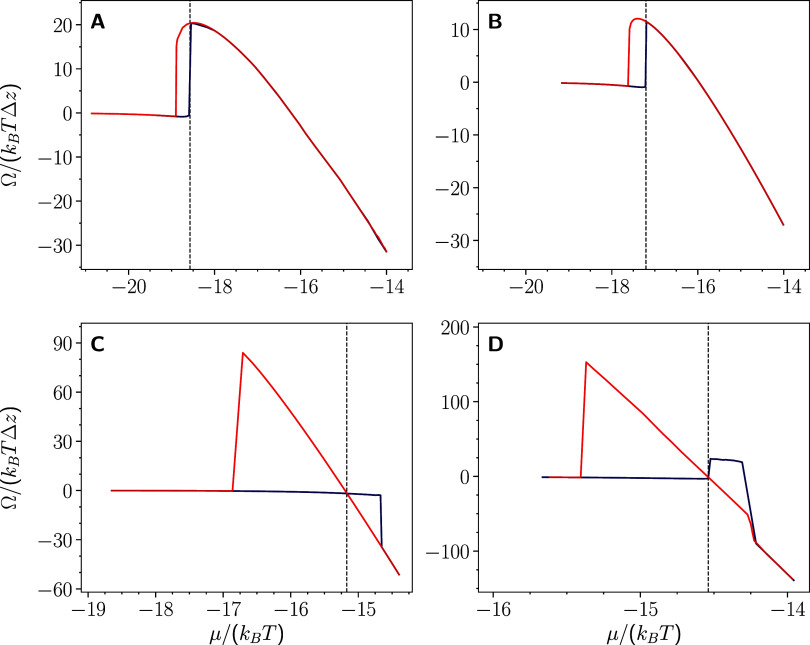
Grand-potential vs chemical potential plots for water adsorbed on carbon slit-pores of (A) 7 Å, (B) 8 Å, (C) 14 Å, and (D) 30 Å width, at 425 K, predicted with the SAFT-VR Mie - cDFT model. The corresponding isotherms are illustrated in Figure 3.

**Figure 5:**
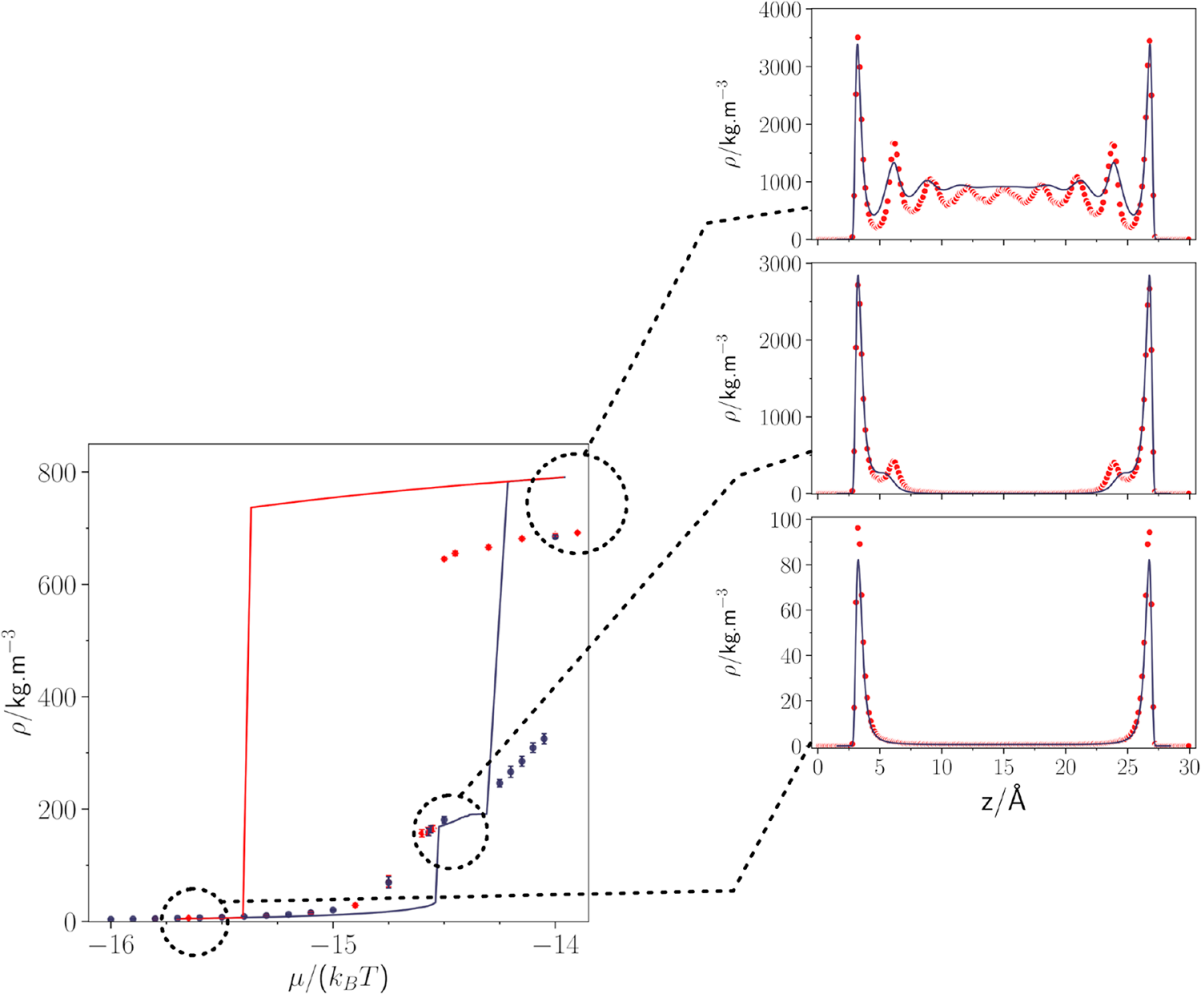
On the left, the adsorption (blue) and desorption (red) isotherms for water on carbon-slit pores of 30 Å width. The density profiles corresponding to three different points of the adsorption isotherms are shown on the right, from the hightest (top) to the lowest (bottom) density. In the middle profile, a second density peak can be seen near the first peak, corresponding to the formation of a second adsorption layer. Symbols: GCMC simulations; Full lines: SAFT-VR Mie - cDFT model.

Malheiro et al.^45^ have also shown that, under similar conditions, cDFT predicts the appearance of hysteresis for some of the pore sizes considered. In their work, hysteresis does not occur below a bulk pressure corresponding to 10% of the saturation bulk pressure, which means that we should not observe metastable phases under the conditions considered in Figure 2A–C. Also, hysteresis is not observed in the 7 Å pore. Nonetheless, the fact that we are considering different molecular models for the fluid, and, as a consequence, different solid–fluid parameters, prevents a direct comparison of the models. For a fairer comparison, the external field imposed by the solid walls on the fluid should be the same, which implies that the solid–fluid parameters in eq 50, σ_cross_, ε_cross_, need to be equal to the ones reported in their paper, ε_cross_/*k*_B_ = 91.717 K and σ_cross_ = 3.2165 Å. A comparison between the results of the two models is presented is Figure 6. The agreement between them is notable, despite the conceptual differences between the models. In the model presented by Malheiro et al.,^45^ the spherical segments interact through a square-well potential. The functional for dispersive interactions is proposed by splitting the first order perturbation term into contributions from short and long-range interactions. While the WDA formalism is used for the former, a mean-field approach is proposed for the latter. In addition, their factorization of the association strength is based on the RDF of a square-well fluid evaluated at contact, whereas we followed the formalism of Yu and Wu,^50^ i.e., the hard-sphere RDF is used instead. The fact that both models exhibit similar behavior for the cases analyzed here indicates that most of the structure of the confined water is determined by the hard-sphere model and by the external field, which is shared by both approaches.

**Figure 6:**
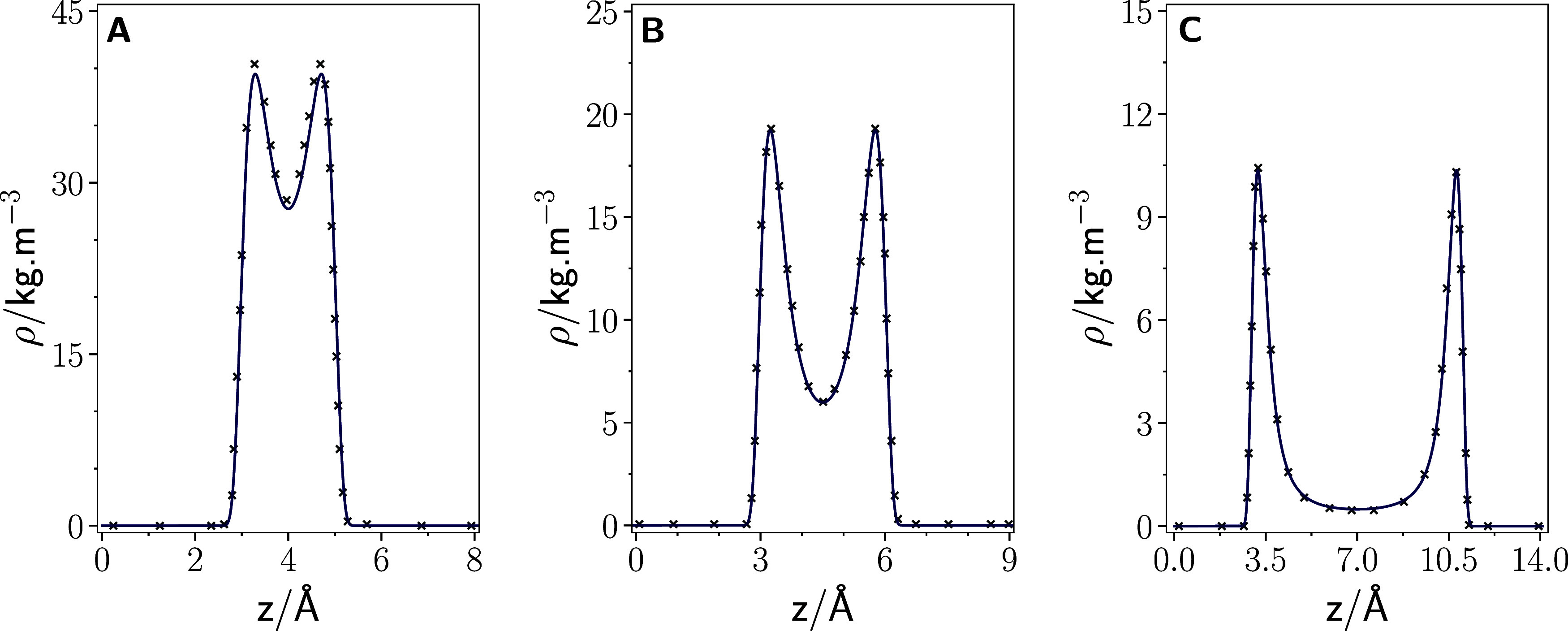
Density profiles of water adsorbed on carbon slit-pores of (A) 8 Å, (B) 9 Å, and (C) 14 Å, at 425 K, with the corresponding bulk density equal to 2.267 kg·m^–3^. Full lines: SAFT-VR Mie - cDFT model; Crosses: NLDFT/SAFT-VR model.^45^

In an attempt to eliminate the influence of the attractiveness of the external potential on the performance of the new functionals, we also have considered water near hard-walls, i.e., purely repulsive walls. In such a case, GCMC and cDFT results are shown in Figure 7 for a 8 Å pore, considering bulk densities of 3.425 kg·m^–3^ (a) and 963.804 kg·m^–3^ (b), as calculated with the SAFT-VR Mie EoS. Small deviations can be observed at the lowest density (Figure 7A), with both cDFT and GCMC results exhibiting a slightly downward curvature. Overall, the density of the confined fluid is close to the bulk density predicted with the EoS. On Figure 7B, the deviations are more pronounced. cDFT generally overestimates the density throughout the pore, with higher deviations shown near the walls. This behavior is consistent with the results observed for the attractive walls (Figures 3 and 5), for which the corresponding isotherms indicate that the average density of the adsorbed fluid, once the liquid-like phase is present, is overestimated in comparison to simulations. Since the WDA formalism is expected to satisfy the contact value theorem,^20^ we believe that the high deviations observed for the contact densities are inherent to the performance of the bulk equation of state in describing the PVT behavior of water under such conditions.

**Figure 7:**
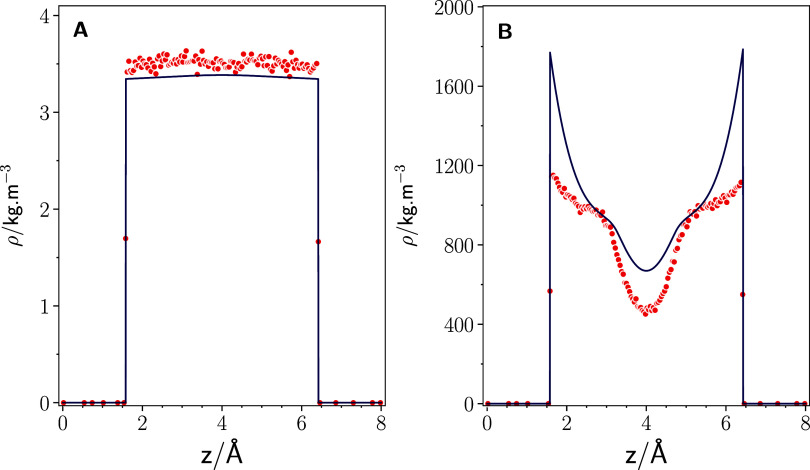
Density profiles of water confined between to parallel hard-walls, separated by 8 Å. The bulk density is equal to 3.425 kg·m^–3^, on (A), and 963.804 kg·m^–3^, on (B). Full lines: SAFT-VR Mie - cDFT model; Symbols: GCMC simulation results obtained with parameters and initial conditions μ* = −13.9665, *L* = 1581.14Å, and ρ^ini^ = 0.00005 Å^–3^ for (A), and μ* = −12, *L* = 84.515 Å, and ρ^ini^ = 0.00175 Å^–3^ for (B).

## Conclusions

The SAFT-VR Mie Equation of State, known for its accurate predictions of thermodynamic properties of bulk fluids, has been extended to inhomogeneous associating fluids confined in slit pores. The analysis of the density profiles of confined water, compared to the results of GCMC simulations, and to another set of results of a similar theoretical model available in the literature, has confirmed the consistency of the approach.

The occurrence of capillary condensation and hysteresis has been addressed by examining the adsorption isotherms on different pore sizes. While cDFT has predicted hysteresis for all the pore sizes considered, GCMC results indicate that it does occur on 7 Å and 8 Å pores. In such cases, we have shown through the grand-potential analysis that the most stable curve is the one representing the adsorption process, which generally agrees to the predictions of GCMC. For 14 Å and 30 Å pores, the loci of the phase transition coincide with the ones identified in the adsorption and desorption curves of GCMC results, respectively. For the 30 Å pore, both cDFT and GCMC predicts the formation of a second adsorption layer, followed by the complete transition to a liquid-like, dense phase. While cDFT indicates the formation of the second layer as a sharp increase in the density of the adsorbed fluid, similarly to the prediction of capillary condensation, GCMC predicts a smoother transition.

There are generally two types of deviations between the results of GCMC and the cDFT model, when it comes to an accurate representation of the phase behavior of water adsorbed on slit pores. The first one is the mismatch between the occurrence of hysteresis, as commented previously. The other is the overestimation of the density of the adsorbed fluid, for a given bulk chemical potential. This is specially true when the fluid in the pores exhibits a liquid-like phase. We attribute such differences to the approximations underlying the development of the functionals for each of the terms of the Helmholtz energy, i.e., the hard-sphere, dispersion, and association contribution. Even for a purely repulsive fluid, it is not expected that the density profiles will be exactly the same. If we consider an adsorption isotherm, which results from the integration of the density profiles under different bulk phase pressures (or chemical potentials), the differences might become even more clear.

A natural extension of this work is to consider confined mixtures of associating fluids. Also, the representation of more realistic porous materials, e.g., zeolites, metal–organic or covalent-organic frameworks, could be addressed by extending the current implementation to 3D geometries. Another possibility regards the reformulation of the functional proposed for the association contribution in such a way to preserve the consistency with the association kernel proposed by Dufal et al.,^32^ i.e., on the basis of the radial distribution function of the Mie fluid, instead of that of the hard-sphere fluid. It is also desirable to evaluate the effect of different numerical procedures on the solutions of the Euler–Lagrange equations, especially for multiple solutions, as in some of the examples treated here.

## Supplementary Material



## Notes

Additional details on the calculations of the derivative of the functional of the associative contributions with respect to the density profile. Details on the acceptance criterion of the trial moves implemented in the GCMC simulations (PDF)
