# Supplementary material for: Classical Density Functional Theory Coupled to the SAFT-VR Mie Equation of State: Extension to Associating Fluids
Source: J Phys Chem B. 2026 May 23;130(22):5712–24. doi: 10.1021/acs.jpcb.6c00630 (PMC13244462; doi:10.1021/acs.jpcb.6c00630)
Supplement: Supplementary file 1 [file jp6c00630_si_001.pdf]

Classical Density Functional Theory coupled to the SAFT-VR Mie Equation of State: extension to associating fluids

André de Freitas Gonçalves, Nathan Barros de Souza, and Luis Fernando Mercier Franco*

Universidade Estadual de Campinas (UNICAMP), Faculdade de Engenharia Química, Campinas, CEP:13083-852, SP, Brazil.

E-mail: lmfranco@unicamp.br

Derivative of the functional for the associating interactions

An expression for the derivative of the functional related to the associating interactions[1] with respect to the density profile can be obtained by applying the chain rule to Equation (42) of the main text:

$$\frac{\delta\beta\mathcal{F}^{\text{assoc}}[\rho(\mathbf{r})]}{\delta\rho(\mathbf{r})} = \int\sum_{\alpha}\frac{\partial\Phi^{\text{assoc}}}{n_\alpha(\mathbf{r'})}\frac{\delta n_\alpha(\mathbf{r'})}{\delta\rho(\mathbf{r})}\mathrm{d}\mathbf{r'}, \tag{S.1}$$

where $\alpha = n_0, n_2, n_3, \mathbf{n}_{V2}$, and Φ^{assoc} is the Helmholtz energy density due to association, which is a function of the weighted densities adapted from the Fundamental Measure Theory (FMT):

$$\Phi^{\text{assoc}}(n_\alpha) = n_0(\mathbf{r})\zeta(\mathbf{r})\sum_{a=1}^{s} n_a\left[\ln\chi_a(\mathbf{r}) - \frac{1}{2}\chi_a(\mathbf{r}) + \frac{1}{2}\right]. \tag{S.2}$$

For water, $s = 2$ and $n_1 = n_2 = 2$ (or $n_a = n_b = 2$), i.e. two association sites for each of the two association types, and the fraction of non-bonded segments reads as

$$\chi(\mathbf{r}) = \frac{-1 + \sqrt{1 + 8n_0(\mathbf{r})\zeta(\mathbf{r})\Delta_{ab}(\mathbf{r})}}{4n_0(\mathbf{r})\zeta(\mathbf{r})\Delta_{ab}(\mathbf{r})}. \tag{S.3}$$

The strength of association, $\Delta_{ab}(\mathbf{r})$, is calculated with Equations 34, 36, and 41 of the main text. It is important to note that both $\chi(\mathbf{r})$ and $\Delta_{ab}(\mathbf{r})$ are functions of the weighted densities, so ordinary derivation rules, e.g. chain and product rules apply, when taking the derivatives of Equations S.2 and S.3 with respect to the weighted densities. Equation S.1 can be rewritten as a sum of convolutions of the derivatives of Equation S.2 with the weight functions of FMT ($\alpha = n_0, n_2, n_3, \mathbf{n}_{V2}$):

$$\frac{\delta\beta\mathcal{F}^{\text{assoc}}[\rho(\mathbf{r})]}{\delta\rho(\mathbf{r})} = \sum_{\alpha}\left(\frac{\partial\Phi^{\text{assoc}}}{n_\alpha(\mathbf{r})} \circledast \omega_i^{\alpha}(\mathbf{r})\right). \tag{S.4}$$

Phase equilibria for bulk water

The performance of the model is also evaluated with respect to the description of the vapor-liquid equilibrium (VLE) for pure water, under bulk conditions. To do so, the external potential of Equation 6 of the main text is reduced to zero, so that the local density reduces to the bulk density throughout the pore. Under such conditions, the derivative of the residual Helmholtz energy with respect to the density profile corresponds to the bulk chemical potential. We search for two distinct density values that corresponds to the same chemical potential. Multiple solutions can be found, but the one that corresponds to the VLE is the global minimum of the grand potential, which is determined as the point in the plot of grand-potential vs. chemical potential where both branches of the curve intercept, as shown in Figure 1B. By performing the analysis for different temperatures, we can obtain the phase diagram for bulk water (Figure 1A). As expected, the results of cDFT for bulk water match the predictions of the SAFT-VR Mie EoS (Figure 7A of Dufal *et al.*[3]), which exhibit

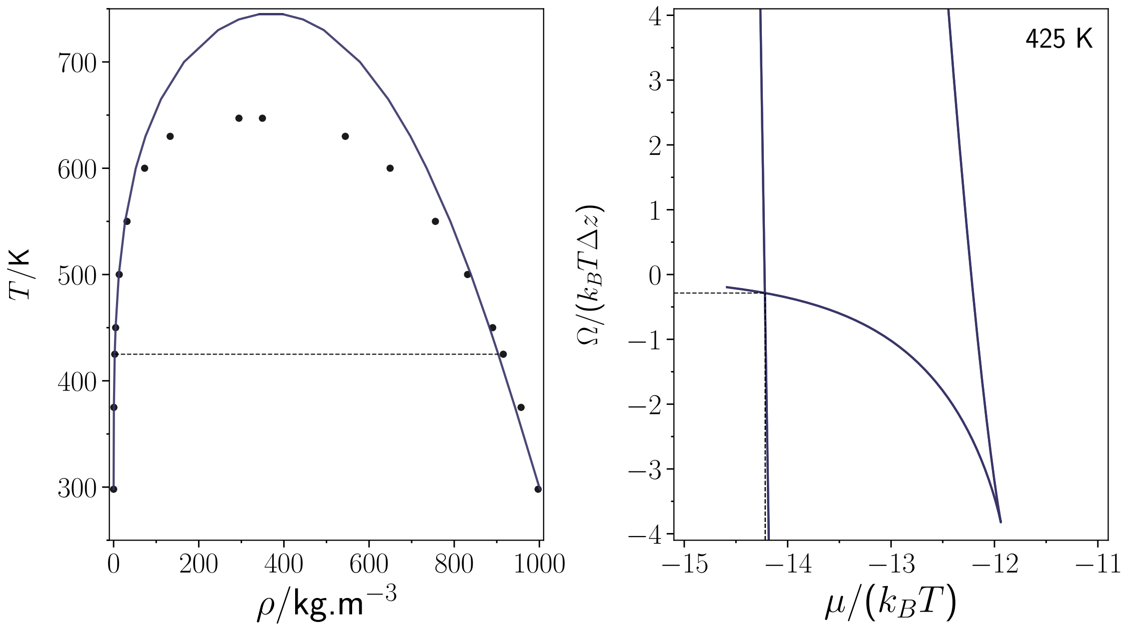

Figure 1: On A, the phase diagram for water obtained with the SAFT-VR Mie-cDFT model (full lines), in comparison to the NIST database (black dots).[2] On B, the grand-potential *vs.* chemical potential plot for the system at 425 K, indicating the locus of the chemical potential corresponding to the vapor-liquid transition.

large deviations near the critical region, but provide good results at 425 K, the temperature considered in our calculations.

GCMC Simulation: Trial moves and Acceptance criterion

Let $\mathbf{r}_p$ be the position of the center of mass of a spherical molecule p with diameter σ_i. We assume a four-site associative model for the water molecule, in which the association sites are located at a distance $r_d = 0.4\sigma_i$ from the molecular center of mass and arranged in a distorted tetrahedral geometry. In this arrangement, the angle θ_{site} between identical sites is equal to 104.5°. Under these assumptions, the positions of the association sites are given

by

$$\mathbf{r}_1^{(p)} = \mathbf{r}_p + \left[r_d \sin\left(\frac{\theta_{\text{site}}}{2}\right), 0, r_d \cos\left(\frac{\theta_{\text{site}}}{2}\right)\right], \tag{S.5a}$$

$$\mathbf{r}_2^{(p)} = \mathbf{r}_p + \left[-r_d \sin\left(\frac{\theta_{\text{site}}}{2}\right), 0, r_d \cos\left(\frac{\theta_{\text{site}}}{2}\right)\right], \tag{S.5b}$$

$$\mathbf{r}_3^{(p)} = \mathbf{r}_p + \left[0, r_d \sin\left(\frac{\theta_{\text{site}}}{2}\right), -r_d \cos\left(\frac{\theta_{\text{site}}}{2}\right)\right], \tag{S.5c}$$

$$\mathbf{r}_4^{(p)} = \mathbf{r}_p + \left[0, -r_d \sin\left(\frac{\theta_{\text{site}}}{2}\right), -r_d \cos\left(\frac{\theta_{\text{site}}}{2}\right)\right], \tag{S.5d}$$

where $\mathbf{r}_1^{(p)}$ and $\mathbf{r}_2^{(p)}$ correspond to the positions of the E sites on molecule p, while $\mathbf{r}_3^{(p)}$ and $\mathbf{r}_4^{(p)}$ correspond to the positions of the H sites.

In the GCMC simulations, four types of moves were considered. These are recalled as follows: (i) translation of the molecule's center of mass, (ii) rotation of the molecule about its center of mass, (iii) insertion of a molecule, and (iv) deletion of a molecule.

In translational trial moves, molecules are displaced by a random vector $\Delta\mathbf{r}$, the components of which are sampled independently from a uniform distribution in the interval $[-\Delta r_{\max}, \Delta r_{\max}]$, where $\Delta r_{\max}$ is the maximum translational displacement. Let $\mathbf{r}_{p,m}$ be the position of the molecule in microstate m before the move is performed. The new position $\mathbf{r}_{p,n}$ of the molecule in microstate n after the translational trial move is then given by

$$\mathbf{r}_{p,n} = \mathbf{r}_{p,m} + \Delta\mathbf{r}, \tag{S.6}$$

where $\Delta\mathbf{r} = \big((2\zeta_x - 1)\Delta r_{\max}, (2\zeta_y - 1)\Delta r_{\max}, (2\zeta_z - 1)\Delta r_{\max}\big)$, with ζ_x, ζ_y, $\zeta_z \in [0, 1)$ being independent, uniformly distributed random numbers associated with the x-, y- e z-directions, respectively.

Following the same translational move, the new positions of the association sites a of molecule p are given by

$$\mathbf{r}_{a,n}^{(p)} = \mathbf{r}_{a,m}^{(p)} + (\mathbf{r}_{p,n} - \mathbf{r}_{p,m})\,. \tag{S.7}$$

In rotational trial moves, molecules are rotated about their center of mass by a random rotation angle θ_r, sampled independently from a uniform distribution in the interval $[-\Delta\theta_{\text{max}}, \Delta\theta_{\text{max}}]$, where $\Delta\theta_{\text{max}}$ is the maximum rotational displacement. Rotational trial moves were implemented using quaternions: a four-component entity consisting of a scalar and a three-dimensional vector.

The rotation quaternions (or unit quaternions) are constructed according to the following scheme:

a) A rotation axis $\hat{\mathbf{v}}_r$ is generated by selecting random points on the surface of a sphere using the Marsaglia's algorithm.[4]

b) Next, a rotation angle is generated as $\theta_r = (2\zeta - 1)\Delta\theta_{\text{max}}$, where $\zeta \in [0, 1)$ is a uniformly distributed random number.

c) Finally, a rotation quaternion is constructed as $\mathbf{q}_r = \cos\left(\frac{\theta_r}{2}\right) + \sin\left(\frac{\theta_r}{2}\right)\hat{\mathbf{v}}_r$. Essentially, a quaternion represents a rotation of θ_r about an axis $\hat{\mathbf{v}}_r$.

In the GCMC simulations, rotational trial moves leave the molecular center of mass unchanged, while modifying the positions of the association sites. Let $\mathbf{r}_a^{\text{ref}}$ be a reference vector defined from the center of mass of molecule p ($\mathbf{r}_p$) to the center of mass of association site a on molecule p in microstate m ($\mathbf{r}_{a,m}^{(p)}$). Thus:

$$\mathbf{r}_a^{\text{ref}} = \mathbf{r}_{a,m}^{(p)} - \mathbf{r}_p. \tag{S.8}$$

To obtain the new positions of the association sites a on molecule p after rotation by the quaternion $\mathbf{q}_r$, the Hamilton product is first applied to the reference vector:

$$\mathbf{r}_a^{\text{rot}} = \mathbf{q}_r \otimes \mathbf{r}_a^{\text{ref}} \otimes \mathbf{q}_r^*, \tag{S.9}$$

where $\mathbf{r}_a^{\text{rot}}$ corresponds to the rotated reference vector and $\mathbf{q}_r^*$ denotes the conjugate of the

rotation quaternion, such that $\mathbf{q}_r^* = \cos\left(\dfrac{\theta_r}{2}\right) - \sin\left(\dfrac{\theta_r}{2}\right)\hat{\mathbf{v}}_r$.

After the rotation, the new positions of the association sites in microstate n are given by

$$\mathbf{r}_{a,n}^{(p)} = \mathbf{r}_p + \mathbf{r}_a^{\mathrm{rot}}. \tag{S.10}$$

In summary:

$$\mathbf{r}_{a,n}^{(p)} = \mathbf{r}_p + \mathbf{q}_r \otimes \left(\mathbf{r}_{a,m}^{(p)} - \mathbf{r}_p\right) \otimes \mathbf{q}_r^*. \tag{S.11}$$

Insertion trial moves of molecule $N+1$ are performed by randomly selecting the position of the center of mass of the new molecule and the positions of its association sites.

Regarding the center-of-mass position, the coordinates of a vector $\mathbf{s}_{N+1} \in [-1/2, 1/2)^3$ are first sampled randomly and independently. This vector represents the scaled position of the new molecule within a unit cube centered at $(0, 0, 0)$.

Next, the coordinates of the unit box are transformed into the simulation box coordinates by performing

$$\mathbf{r}_{N+1} = \mathbf{s}_{N+1} \cdot \mathbf{B}, \tag{S.12}$$

where $\mathbf{B}$ corresponds to the (orthorhombic) simulation box, represented by the diagonal matrix:

$$\mathbf{B} = \begin{bmatrix} L & 0 & 0 \\ 0 & L & 0 \\ 0 & 0 & H \end{bmatrix}, \tag{S.13}$$

where H is the pore width along the z-direction and L is the pore length along the x- e y-directions, corresponding to a square cross-sectional area.

The positions of the association sites are initially given by Eq. S.5, using the center of

mass $\mathbf{r}_{N+1}$ of the new molecule as a reference:

$$\mathbf{r}_1^{(N+1)} = \mathbf{r}_{N+1} + \left[r_d \sin\left(\frac{\theta_{\text{site}}}{2}\right), 0, r_d \cos\left(\frac{\theta_{\text{site}}}{2}\right) \right], \tag{S.14a}$$

$$\mathbf{r}_2^{(N+1)} = \mathbf{r}_{N+1} + \left[-r_d \sin\left(\frac{\theta_{\text{site}}}{2}\right), 0, r_d \cos\left(\frac{\theta_{\text{site}}}{2}\right) \right], \tag{S.14b}$$

$$\mathbf{r}_3^{(N+1)} = \mathbf{r}_{N+1} + \left[0, r_d \sin\left(\frac{\theta_{\text{site}}}{2}\right), -r_d \cos\left(\frac{\theta_{\text{site}}}{2}\right) \right], \tag{S.14c}$$

$$\mathbf{r}_4^{(N+1)} = \mathbf{r}_{N+1} + \left[0, -r_d \sin\left(\frac{\theta_{\text{site}}}{2}\right), -r_d \cos\left(\frac{\theta_{\text{site}}}{2}\right) \right]. \tag{S.14d}$$

Subsequently, a random rotation quaternion $\mathbf{q}_r$ is constructed, in the same manner as for rotational trial moves, except that the rotation angle θ_r is now sampled from the interval $[-\pi, \pi)$. This procedure ensures uniform sampling of molecular orientations and preserves detailed balance in the insertion move. Finally, by applying the Hamilton product, the positions of the association sites are transformed by $\mathbf{q}_r$, such that

$$\mathbf{r}_a^{(N+1)} \rightarrow \mathbf{r}_{N+1} + \mathbf{q}_r \otimes \left(\mathbf{r}_a^{(N+1)} - \mathbf{r}_{N+1} \right) \otimes \mathbf{q}_r^*. \tag{S.15}$$

Both translational and rotational trial moves are sampled in the canonical (NVT) ensemble, for which the acceptance probability associated with a transition from microstate m to microstate n is determined by the value of:

$$\mathscr{P}_{mn} = \exp(-\beta \delta \mathcal{V}_{mn}), \tag{S.16}$$

where $\beta = 1/(k_\text{B}T)$, with k_B being the Boltzmann constant and T the system temperature.

By contrast, insertion and deletion trial moves are sampled in the grand canonical (μVT),

for which the acceptance probability is given by

$$\mathscr{P}_{mn} = \exp(-\beta\delta\mathcal{V}_{mn})\exp(\beta\mu)\frac{V}{\Lambda_i^3(N+1)} \qquad \text{(insertion)}, \tag{S.17a}$$

$$\mathscr{P}_{mn} = \exp(-\beta\delta\mathcal{V}_{mn})\exp(-\beta\mu)\frac{N\Lambda_i^3}{V} \qquad \text{(deletion)}, \tag{S.17b}$$

where μ is the chemical potential, V is the simulation box volume, and N is the number of molecules in microstate m, i.e., prior to the insertion/deletion trial move.

The quantity Λ_i is the thermal de Broglie wavelength of species i, defined as

$$\Lambda = \sqrt{\frac{h^2}{2\pi m_i k_\mathrm{B} T}}, \tag{S.18}$$

where h is Planck's constant and m_i is the mass of a molecule of species i.

In Eq. S.16 and S.17, $\delta\mathcal{V}_{mn}$ denotes the total potential energy difference between microstates m and n. For translational and rotational trial moves, $\delta\mathcal{V}_{mn}$ is computed from the change in the potential energy of the displaced molecule p (see the main text for explicit expressions). If molecule p is initially bonded to a molecule k, a bond rupture induced by the trial move triggers a reassociation check (see the main text for details). When molecule k reassociates with a neighboring molecule $q \neq p$, the resulting increase in potential energy is also included explicitly in $\delta\mathcal{V}_{mn}$.

For insertion trial moves, $\delta\mathcal{V}_{mn}$ corresponds to the increase in the total potential energy upon adding molecule $N+1$, such that $\delta\mathcal{V}_{mn} = \phi_{N+1}$. Insertion trial moves do not trigger reassociation events. For deletion trial moves, $\delta\mathcal{V}_{mn}$ corresponds to the decrease in the total potential energy upon removing molecule p, such that $\delta\mathcal{V}_{mn} = -\phi_p$. If the deletion of molecule p induces a bond rupture followed by a reassociation event of molecule k, the resulting increase in the potential energy of the affected molecule k is explicitly included in $\delta\mathcal{V}_{mn}$.

Finally, in Eqs. S.16 and S.17, the trial move is accepted if $\mathscr{P}_{mn} \geq \zeta$, where $\zeta \in [0,1)$ is

a uniformly distributed random number; otherwise, the move is rejected.

References

(1) Yu, Y.-X.; Wu, J. A fundamental-measure theory for inhomogeneous associating fluids. *The Journal of Chemical Physics* **2002**, *116*, 7094–7103.

(2) National Institute of Standards and Technology *Thermophysical Properties of Fluid Systems - NIST Chemistry WebBook, SRD 69*; 2026; [Online; Available from `https://webbook.nist.gov/cgi/cbook.cgi?ID=C7732185`; Accessed 28-March-2026].

(3) Dufal, S.; Lafitte, T.; Haslam, A. J.; Galindo, A.; Clark, G. N.; Vega, C.; Jackson, G. The A in SAFT: developing the contribution of association to the Helmholtz free energy within a Wertheim TPT1 treatment of generic Mie fluids. *Molecular Physics* **2015**, *113*, 948–984.

(4) Marsaglia, G. Choosing a point from the surface of a sphere. *The Annals of Mathematical Statistics* **1972**, *43*, 645–646.
